# Bone Health in Men with Prostate Cancer: Review Article

**DOI:** 10.1007/s11914-019-00536-8

**Published:** 2019-11-23

**Authors:** Salma A M El Badri, Abdulazeez Salawu, Janet E Brown

**Affiliations:** 1 Weston Park Hospital, Sheffield, UK; 2 Department of Oncology and Metabolism, University of Sheffield, Sheffield, UK

**Keywords:** Prostate cancer, Bone health, Osteoporosis, Androgen deprivation therapy

## Abstract

**Purpose of Review:**

The improvement in prostate cancer survival over time, even in those with advanced disease, has led to an increasing recognition of the impact of prostate cancer and its treatment on bone health. Cancer treatment–induced bone loss (CTIBL) is a well-recognized entity but greater awareness of the risks associated with CTIBL and its treatment is required.

**Recent Findings:**

The principal culprit in causing CTIBL is hormonal ablation induced by prostate cancer treatment, including several new agents which have been developed in recent years which significantly improve survival, but may cause CTIBL. This review discusses the impact of prostate cancer and its treatment on bone health, including published evidence on the underlying pathophysiology, assessment of bone health, and strategies for prevention and treatment.

**Summary:**

It is important to recognize the potential cumulative impact of systemic prostate cancer treatments on bone health.

## Introduction

Prostate cancer is the second most commonly diagnosed cancer in men with an estimated 1.3 million cases diagnosed in 2018 according to the most recent International Agency for Research on Cancer (IARC) report [1]. Men diagnosed with prostate cancer are now living longer. Prostate cancer survival has tripled in the last 40 years in the UK, with about 84% of men surviving their disease for ten years or more (2010–2011) [2]. In the USA, prostate cancer mortality has declined by 51% from 1993 to 2016 [3]. This improved survival is mainly attributed to advances in treatment, with some dispute about the impact of screening and earlier detection on mortality [4]. As patients are now living with prostate cancer for longer, the long-term impact of prostate cancer and its treatment on bone health in men is increasingly recognized.

**Fig. 1:**
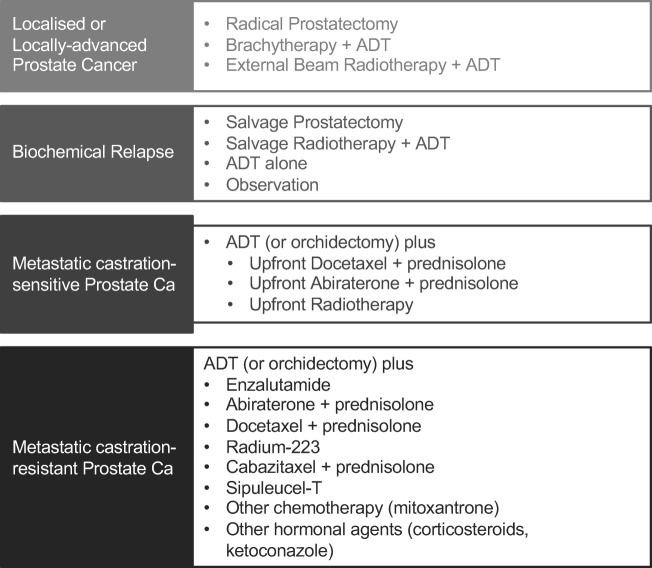
Treatment of prostate cancer at different stages

Androgens and the Androgen Receptor (AR) signalling pathway play a key role in prostate cancer pathophysiology. Androgen deprivation therapy (ADT), which can be achieved surgically (by orchidectomy) or chemically using luteinizing hormone-releasing hormone (LHRH) agonists and LHRH antagonists is therefore a cornerstone in the treatment of prostate cancer (Fig. 1). ADT is used in prostate cancer treatment at various stages: in men who present with or progress to metastatic disease; men who receive radical radiotherapy for localized or locally advanced disease and men who progress on a period of watchful waiting and are not fit for radical treatment [5–8]. Novel means for hormonal manipulation such as androgen synthesis inhibitors or AR signalling inhibitors are utilised in addition to ADT in the advanced disease setting.

Long-term ADT has well-recognized negative impact on bone mineral density and increases fracture risk in men [9–12]. A large observational study conducted by Shahinian et al. looked at the outcomes in 50,613 patients with prostate cancer. Of those who survived for at least 5 years after diagnosis, data showed that 19.4% of those who received ADT sustained a fracture, compared to 12.6% of those who did not receive ADT (*p* < 0.001) [13]. This negative impact on bone health also applies to other prostate cancer treatments including chemotherapy, glucocorticoids and novel hormone manipulation agents. These are used, in addition to background ADT, for the treatment of advanced prostate cancer, which involves bone in an estimated 90% of cases [14] with significant potential for morbidity and skeletal-related events (SREs) such as pathological fractures, pain, spinal cord compression and need for radiotherapy.

There is also evidence to suggest that even before initiating ADT, men with advanced prostate cancer have a higher incidence of osteoporosis and osteopenia compared to age-matched controls [15]. A separate population-based cohort study showed that men who have a high baseline risk of skeletal complications developed more fractures after initiating ADT [16]. Osteoporotic fractures in men with prostate cancer have been shown to correlate with poor survival outcomes [16, 17]. In addition, osteoporotic fractures also have a significant socio-economic impact. A report published by Hernlund et al. on osteoporosis in the European Union (EU) revealed that there were 3.5 million new fragility fractures in the EU in 2010 with an economic burden estimated at 37 billion euros and that this is expected to rise by 25% in 2025 [18].

Clinical guidelines from the National Institute for Health and Care Excellence (NICE) in the UK recommend that fracture risk is considered for all men receiving ADT and that treatment is offered to all those found to have osteoporosis [19]. Similarly, the European Association of Urology (EAU), European Society for Radiotherapy and Oncology (ESTRO) and International Society for Geriatric Oncology (SIOG) guidelines suggest that BMD assessment should be performed prior to the initiation of long-term ADT [20].

In this review, we will discuss current molecular and clinical understanding of the impact of metastatic disease and cancer treatment–induced bone loss (CTIBL) on bone health in prostate cancer patients and its management.

## Pathophysiology

### Prostate Cancer Bone Metastases

Bone is the most common site of metastasis from prostate cancer as shown in an autopsy study of 1589 patients with prostate cancer in which 90% were found to have bone involvement [14]. Bone metastases are associated with an increased morbidity and a negative impact on quality of life mainly through SREs [21]. Treatment strategies are therefore directed at delaying the onset of SREs and hence preserving the quality of life and functional status in this patient group [22].

The exact mechanisms for development of bone metastases in prostate cancer patients remain unclear and studies are ongoing in this field. The bone microenvironment, however, is recognized as a significant mediator of prostate cancer bone tropism and this is mediated by the CXCL16/CXCR6 axis. Circulating tumour cells migrate towards the bone based on a gradient of chemokines and ligands released by the bone marrow. These tumour cells then parasitize the bone microenvironment for haematopoietic stem cells (HSC) and become dormant in the bone marrow. It is therefore suggested that a specific component of the bone marrow microenvironment can serve as a potential therapeutic target in prostate cancer patients with bone metastases [23].

RANKL is a major mediator of normal bone remodelling and binds to its receptor RANK on the surface of osteoclast progenitors, resulting in osteoclast differentiation and bone resorption. Disseminated prostate cancer cells enhance RANKL expression on osteoblasts by secreting parathyroid hormone-related protein leading to osteoclastogenesis and increased bone resorption, which in turn creates space for tumour cells to grow within the bone marrow [24].

Further studies looking at the specific molecular mechanisms controlling the formation and progression of bone metastases in prostate cancer patients are important as they can set new targets for the development of novel therapies in this patient group.

### Cancer Treatment–Induced Bone Loss

The role of sex steroids on bone homeostasis has been extensively studied and, in recent years, the development of mouse models with global and cell-specific deletions in Oestrogen and Androgen Receptors (ERα, ERβ, AR) has evolved our understanding of this role [25–28]. Androgen receptor (AR) signalling in osteoblasts is responsible for the protective effects of androgens on trabecular bone mass, leading to a decrease in osteoclast numbers and bone resorption. Oestrogens, produced via aromatization of androgens in males, protect against endocortical resorption, at least in part, via ERα signalling in mesenchymal/stromal cells [26]. Oestrogens play an important role in regulating the RANKL/RANK/OPG pathway, which influences osteoclast activity and has important therapeutic implications [26–29]. Collective evidence from several interventional and observational human studies supports the theory that oestrogen plays a much more significant role in regulating bone metabolism in men than testosterone [30–33].

Following initiation of ADT, the levels of both testosterone and oestradiol fall rapidly and significantly, leading to disruption of bone integrity. A prospective study conducted at an academic medical centre in the USA investigated the rate of bone loss following initiation of ADT in men with prostate cancer. This showed the reduction in bone mineral density to be most significant in the first 12 months after initiation of ADT; hence, the importance of early initiation of preventative measures [34]. It also showed that the rate of bone loss in prostate cancer patients initiating ADT was 5- to 10-fold higher than in either healthy age-matched controls or men with prostate cancer with normal hormone levels [34]. This deleterious effect on bone health is directly related to the duration of androgen deprivation. A progressive decline in bone mineral density was observed, even up to 10 years, with prolonged use of ADT in a separate cross-sectional study, and this was more pronounced with continuous ADT and surgical castration compared to intermittent ADT [35].

**Fig. 2:**
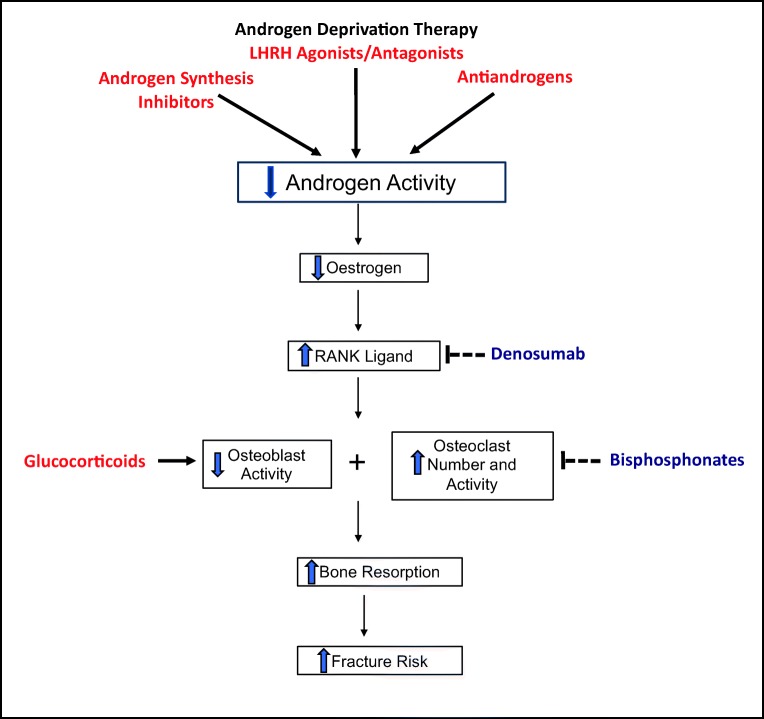
Mechanistic role of androgen deprivation therapy

Glucocorticoid-induced bone loss is well-recognized and mediated through increased apoptosis of osteoblasts and osteocytes, impaired differentiation of osteoblasts and increased life span of osteoclasts [36]. Glucocorticoids are an important component of the treatment of metastatic prostate cancer where they are often used in conjunction with chemotherapy; with adrenal synthesis inhibitors (such as abiraterone); or sometimes at a low dose as monotherapy. The resultant increase in fracture risk should therefore be taken into consideration when used in these settings [37, 38]. Studies are needed to investigate the potential cumulative effect on bone health when combining ADT with chemotherapy and glucocorticoids. Enzalutamide, a novel oral androgen receptor signalling inhibitor, now approved for use in patients with metastatic castration-resistant prostate cancer, has been shown to be associated with a smaller change in bone mineral density when compared to the effect caused by leuprolide [39••, 40]. Apalutamide is a next-generation non-steroidal androgen receptor antagonist being studied in patients with metastatic castration-resistant prostate cancer and further results and analysis would be needed to investigate its impact on bone health [41••] (Fig. 2).

### Assessment of Bone Health in Prostate Cancer Patients

There is an inverse relationship between fracture risk and BMD with an approximately two-fold increase in fracture risk with each standard deviation reduction in BMD [42, 43]. Dual energy X-ray absorptiometry (DXA) is the most common method of assessing bone mineral density (BMD). Specific skeletal locations are typically measured, including the proximal femur (total hip or femoral neck) and lumbar spine [44, 45]. Measurements are usually reported as a T-score and osteoporosis is defined as a T-score ≥ 2.5 standard deviation below the mean value for young healthy adults [46].

While highly specific, DXA assessment of BMD has a low sensitivity for prediction of fractures with many fragility fractures occurring in individuals who have a non-osteoporotic BMD. Many individuals who sustain a fracture are subsequently found to have non-osteoporotic BMD [47]. Several other factors therefore contribute to fracture risk including advanced age, sex, falls risk, history of previous fractures, family history of fractures, and other lifestyle factors. Nevertheless, DXA remains the gold standard for assessing bone mineral density in this population.

FRAX® (http://www.shef.ac.uk/FRAX) is a risk assessment tool that has been developed to incorporate BMD measurements in addition to these variables for more accurate prediction of fracture risk. It calculates the 10-year probability of a major osteoporotic fracture and of hip fracture [48, 49]. Launched in 2008, it is now the most widely used risk assessment tool in clinical practice having been approved by both the FDA and NICE [49]. Other tools such as QFracture (http://www.qfracture.org), which is based on a UK prospective open cohort of over 2 million patients have also been developed and validated for clinical use [50, 51].

### Prevention and Treatment of CTIBL

Systemic anticancer therapies as well as bone-targeted agents such as zoledronic acid, denosumab and radium-223 have proven effective for the prevention of skeletal-related events from prostate cancer bone metastases and this has been the subject of previous extensive review [52] and is beyond the remit of this article. With improved survival of patients living with bone-metastatic prostate cancer however, the impact of CTIBL continues to grow and is gaining recognition. Several strategies have therefore been evaluated to prevent and treat CTIBL.

### Awareness and Education

Studies have consistently shown that patients often lack knowledge about the risk of CTIBL and means for prevention and treatment [53–55]. There also appears to be a discrepancy between what physicians assume that patients know and what the patients’ perception is about their bone health [56]. It is also interesting that despite physicians’ good knowledge about bone health, there appears to be inadequate adherence to guidelines for screening, monitoring and treatment of CTIBL [57, 58].

Results from a phase 2 study evaluating two education-based models, incorporating patient pamphlets, involvement of family physicians and Bone Health Care Coordinators, to improve bone health care in men receiving ADT have shown these to be feasible and they were associated with improved requesting of baseline Bone Mineral Density (BMD) scans [59••]. In many cases, it would be possible for the family physicians to play a key role in the treatment and monitoring of these patients given their experience in non-cancer-related osteoporosis. In addition, given that the internet is the most widely used source of information for patients, the development of approved online educational tools/websites is thought to be beneficial in raising awareness and adherence with healthy bone behaviour [60].

### Lifestyle Modification

Osteoporosis and osteopenia are common in men with prostate cancer even before initiating ADT, particularly in the elderly population. Several studies have identified factors that affect bone loss in this patient group, including maintenance of high BMI, weight-bearing exercise, avoidance of alcohol and smoking, and possibly high dietary calcium intake could help reduce bone loss [61–64]. However, further studies are required to objectively quantify the impact of these interventions on BMD.

On the basis of potential roles in prostate cancer pathogenesis, calcium and vitamin D supplementation in men with prostate cancer has been a subject of several studies [65–68]. However, no trials to date have evaluated the risk-benefit ratio of calcium and vitamin D supplementation in men receiving ADT. Notably, a systematic review of 12 clinical trials in men with prostate cancer undergoing ADT showed that the currently recommended doses of calcium and vitamin D supplementation for prevention of osteoporosis are inadequate in preventing BMD loss in this group [68]. Further studies are therefore needed to determine the safety and efficacy of higher doses in this population.

Another important consequence of exposure to ADT is sarcopenia, a degenerative loss of muscle mass that is in turn associated with frailty and increased falls risk [69]. When combined with the effects of ADT on BMD, these patients are at an even higher fracture risk with potentially life-threatening complications [70]. Measures such as muscle-strengthening exercise and maintaining healthy nutrition with adequate protein intake have been evaluated and shown to potentially help ameliorate the risk of sarcopenia and its attendant consequences in patients on ADT [71].

### Bone-Targeted Agents

#### Bisphosphonates

The role of bisphosphonates in reducing BMD loss in men with prostate cancer receiving ADT has been extensively studied in several randomized controlled trials (RCTs) (summarised in Table 1). Among the largest of these was the RADAR study conducted by Denham et al. who enrolled 1071 men receiving radical radiotherapy for locally advanced prostate cancer and had in addition, 6 or 18 months of ADT with or without zoledronic acid (4 mg q3-monthly for 18 months) [73]. They found that compared with baseline DEXA measurements, BMD at the hip measured at 4 years was reduced with both 6 and 18 months of ADT (1.7% and 3.7%, respectively; *p* < 0.01) and this BMD reduction was prevented by concomitant administration of zoledronate. There was however, no significant difference in the primary endpoint of incidence of vertebral fractures seen although this was attributed to insufficient sample size and duration of follow-up [86].

**Table 1:** Summary of RCTs of bisphosphonates in men receiving ADT

Study	Study population	*N*	Study groups	Follow-up	Key findings
Smith et al. 2001 [72]	advanced or recurrent PCa and no bone metastases	47	ADT only vs ADT + Pam	48 weeks	-ADT only arm: decrease in BMD of LS (− 3.3%), trochanter (− 2.1%), and total hip (− 1.8%), *p* < 0.001 for all. -No significant change in mean BMD at any skeletal site in ADT + Pam arm.
Michaelson et al. 2007 [73]	Non-metastatic PCa	40	ADT + placebo vs ADT + Zol	12 months	-Increase in BMD in Zol arm compared to placebo in both LS (4% vs − 3.1%, *p* < 0.001) and total hip (0.7% vs − 1.9%, *p* = 0.004), with suppression of serum NTP levels.
Bhoopalam et al. 2009 [74]	Non-metastatic PCa on ADT for ≤ 1 year or > 1 year	93	ADT + placebo vs ADT + Zol	12 months	-Increase in LS BMD in Zol arm seen in both groups (ADT ≤ 1 year: 5.95% in Zol arm vs − 3.23% in placebo arm [*p* = 0.0044], ADT > 1 year: 6.08% in Zol arm vs 1.57% in placebo arm [*p* = 0.0005]), including patients with multiple risk factors for osteoporosis.
Smith et al. 2003 [75]	Non-metastatic PCa	106	ADT + placebo vs ADT + Zol	12 months	-Increase in LS BMD in Zol arm compared to placebo arm (5.6% vs − 2.2%, *p* < 0.001)
Magno et al. 2005 [76]	Locally advanced PCa with osteoporosis at baseline	60	MAB vs MAB + Ner vs Bicalutamide vs Bicalutamide + Ner	12 months	-MAB only arm: significant loss in BMD of LS (− 4.9%, *p* = 0.002) and total hip (− 1.9%, *p* = 0.004). -MAB + Ner arm: no significant BMD change. -Bicalutamide arm: no significant BMD change. -Bicalutamide + Ner arm: increase in LS (+ 2.5%) and total hip (+ 1.6%) BMD—both *p* < 0.05.
Ryan et al. 2006 [77]	PCa without bone metastases, on ADT for ≤ 12 months	120	ADT + placebo vs ADT + Zol	12 months	-Zol arm: increase in femoral neck, total hip, and LS BMD by 3.6% (*p* = 0.0004), 3.8% (*p* < 0.0001), and 6.7% (*p* < 0.0001) respectively.
Ryan et al. 2007 [78]	PCa with or without bone metastases, on ADT for ≤ 12 months	42	ADT + placebo vs ADT + Zol	12 months	-After excluding BMD data from sites of known metastases, patients in the Zol arm had a relative increase in BMD compared to placebo, at the femoral neck (4.2%, *p* = 0.001) and LS (7.1%, *p* < 0.001).
Klotz et al. 2013 [79]	Non-metastatic PCa	186	ADT + placebo vs ADT + Alen	12 months	-Increase in LS BMD in Alen arm compared to placebo (1.7% vs − 1.9%, *p* < 0.0001).
Choo et al. 2013 [80]	Non-metastatic PCa, undergoing RT + 2–3 years of ADT	104	ADT + placebo vs ADT + Ris	24 months	-Non-significant decrease in BMD loss in Ris arm at 2 years compared to placebo.
Greenspan et al. 2007/2008 [81, 82]	Non-metastatic PCa	112	ADT + placebo vs ADT + Alen, crossover at 12 months	24 months	-ADT + Alen arm: increase in BMD of LS by 3.7% (*p* ≤ 0.001) and femoral neck by 1.6% (*p* = 0.008) at 1 year. -At crossover, those continuing Alen had additional BMD gains at both LS and hip, both *p* < 0.01; those who switched to placebo maintained BMD at LS and hip but had BMD loss at radius - *p* < 0.01.
Rodrigues et al. 2007 [83]	PCa patients who had prostatectomy and rising PSA	94	Placebo vs Clo vs Zol	36 months	Placebo arm: mean BMD loss of − 1.82. Clo arm: mean BMD loss − 0.72. Zol arm: mean BMD loss − 0.82.
Israeli et al. 2007 [84]	Locally advanced PCa during first year of ADT	213	ADT + placebo vs ADT + Zol	12 months	-Mean BMD percentage differences were 6.7% for LS and 3.7% for total hip (*p* < 0.0001 for both).
Kachnic et al. 2013 [85]	high grade and/or locally advanced, non-metastatic PCa receiving ADT + RT	96	Zol vs observation	36 months	Increase in BMD in LS (6% vs − 5%, *p* < 0.0001), left total hip (1% vs − 8%, *p* = 0.0002), and left femoral neck (3% vs − 8%, *p* = 0.0007) in Zol arm compared to observation arm.
Denham et al. 2014 [86]	Locally advanced PCa	1071	ADT for 6 months before RT ± additional 12 months ADT ± 18 months Zol	3 years	-Incidence of vertebral fractures was not increased by 18 months compared to 6 months ADT and was not affected by addition of Zol. -Incidence of non-vertebral fractures was significantly related to ADT duration (*p* = 0.013) but not to the addition of Zol.
Taxel et al. 2010 [87]	Locally advanced PCa	40	Placebo vs weekly risedronate	6 months	-The Ris group had no change in femoral neck or total hip BMD, while the placebo group decreased by 2% (*p* = 0.004) and 2.2% (*p* = 0.001), respectively. -The Ris group had an increase in LS BMD of 1.7% from baseline (*p* = 0.04), with no change in the placebo group.
Casey et al. 2010 [88]	Non-metastatic PCa	200	ADT + Zol for 24 months vs ADT alone for 24 months vs ADT alone for 12 months crossing over to ADT + Zol for 12 months.	24 months	-Significant BMD differences between patients receiving ADT alone and ADT + Zol were observed at the 12 months (*p* < or = 0.01 for each site) and 24 months (*p* < 0.05 for each site). -Initiating Zol after 12 months of ADT alone provided BMD benefits but was insufficient to completely restore BMD.
Kapoor et al. 2011 [89]	Non-metastatic PCa with osteoporosis or osteopenia	41	ADT + placebo vs ADT + Zol	12 months	-The change in vertebral BMD in the Zol group (+ 7.93%) was significantly greater (*p* < 0.05) than the change in the placebo group (+ 0.82%).

*RCT* randomized controlled trial, *PCa* prostate cancer, *Pam* pamidronate, *Zol* zoledronate, *BMD* bone mineral density, *LS* lumbar spine, *PF* proximal femur, *NTP* N-telopeptide (a bone turnover marker), *MAB* maximum androgen blockade, *Ner* neridronate, *Alen* alendronate, *RT* radiotherapy, *Ris* risedronate, *Clo* clodronate

Their results however confirmed those from several prior smaller studies [72–74, 77] that all demonstrated a benefit with bisphosphonates in reducing BMD loss among prostate cancer patients on ADT. It is important to note however, that variations in trial design, agents investigated and the heterogeneous populations included in the studies makes direct comparison of their results difficult. In addition, the primary endpoint in most of these studies was the BMD change rather than the incidence of fractures, which is of greater clinical relevance.

Serpa Neto et al. carried out a 2012 meta-analysis of 15 randomized controlled trials on the effects of bisphosphonates in men with prostate cancer treated with ADT [90]. The authors concluded that the use of bisphosphonates had a substantial effect in prevention of fractures (Risk Ratio (RR) 0.80; *p* = 0.005) and osteoporosis (RR 0.39; *p* < 0.00001), without causing any major side effects [90]. This analysis however included two large RCTs of patients with metastatic CRPC, which makes assessing the impact of bisphosphonates specifically on benign fractures difficult. No bisphosphonate is currently licensed for prevention of BMD loss or fractures in prostate cancer patients on ADT.

#### Denosumab

There are relatively few trials evaluating the effect of denosumab on BMD and fracture risk in men with non-metastatic prostate cancer receiving ADT. The most important of these is a large double-blind RCT by Smith et al. that randomized 1468 patients to receiving 6-monthly denosumab injections (60 mg subcutaneously for 3 years) or placebo. The results demonstrated a significant increase in BMD of the lumbar spine at 24 months in the denosumab group compared to placebo (+ 5.6% vs − 1.0%, *p* < 0.001), with a decrease in the incidence of new vertebral fractures at 36 months (1.5% vs 3.9% with placebo, HR 0.38; 95% confidence interval, 0.19 to 0.78; *p* = 0.006). These beneficial effects were observed as early as 1 month and were sustained at 36 months [91].

A comparison of the effects of denosumab and alendronate on BMD and fracture risk was performed in a randomized study of 234 prostate cancer patients on ADT. The authors found that denosumab was associated with a significant increase in BMD (measured at the lumbar spine) at 24 months compared to alendronate (5.6% vs 1.1%, *p* < 0.001) with a concomitant lower incidence of vertebral fractures, which was not statistically significant [92]. Denosumab is currently the only agent that has regulatory approval for the treatment of bone loss associated with hormone ablation in men with prostate cancer at increased risk of fractures.

### Endocrine Agents

The effect of selective oestrogen receptor modulators such as toremifene and raloxifene in prevention of BMD loss in men receiving ADT has also been investigated given the growing recognition of the role of oestrogen in bone metabolism in men. A randomized controlled trial of 48 men with non-metastatic prostate cancer on ADT showed that the addition of raloxifene significantly increased the BMD at the hip (*p* < 0.001) and tended to increase the BMD at the spine (*p* = 0.07) [93]. A larger study of 646 men with prostate cancer on ADT showed that toremifene was associated with a significant relative risk reduction in the incidence of new vertebral fractures of 50% (*p* = 0.05), with a significant increase in BMD at the lumbar spine, hip and femoral neck compared to placebo (*p* < 0.0001). However, more venous thromboembolic events occurred in the toremifene arm [94]. Neither agent is currently licensed for this indication.

## Conclusion and Future Directions

Prostate cancer patients are now living longer, and many patients receive several lines of therapy, which can have a cumulative impact on bone health over a period of years. Early recognition and optimization of bone health is therefore important in this patient group. A number of new agents have been approved and licensed for treatment of prostate cancer in recent years, and more agents are under development, like apalutamide, which will further extend the treatment options available once licensed, and may impact on bone health.

There is a need to raise awareness among patients about the risks of CTIBL as well as developing models to assist physicians to adhere to guidelines for screening and treatment. Several lifestyle modifications have been investigated but in order to objectively quantify their impact on BMD, further studies are required in this patient group. Bisphosphonates have been shown to reduce BMD loss in prostate cancer patients receiving ADT, however, few studies have investigated reduction in fractures and further larger studies are needed in this area.. Denosumab is the only agent that has shown a significant impact on fracture incidence in this patient population and is currently recommended for treatment of CTIBL associated with androgen deprivation therapy.
